# Supplementation with a polyphenolic blend improves post-exercise strength recovery and muscle soreness

**DOI:** 10.3402/fnr.v59.30034

**Published:** 2015-12-18

**Authors:** Kelli A. Herrlinger, Diana M. Chirouzes, Michael A. Ceddia

**Affiliations:** Kemin Foods, LC, Des Moines, IA, USA

**Keywords:** strength, catechins, theaflavins, cortisol, creatine phosphokinase, antioxidant, delayed onset muscle soreness

## Abstract

**Background:**

Exercise can initiate a cascade of inflammatory and oxidative stress–related events leading to delayed onset muscle soreness. Polyphenols possess antioxidant and anti-inflammatory properties.

**Objective:**

The current study examined the effects of a proprietary polyphenolic blend (PB), containing catechins and theaflavins, on exercise performance and recovery following an eccentric exercise challenge.

**Design:**

Male participants (18–35 years of age) received placebo or PB at a low dose (PB-L, 1,000 mg/d) or high dose (PB-H, 2,000 mg/d) for 13 weeks. During the 13th week of supplementation, participants completed an eccentric exercise (40 min downhill treadmill run) followed by a strength assessment (peak torque on isokinetic leg extensions) pre-exercise, and 24, 48, and 96 h post-exercise. Muscle soreness (subjective questionnaire), markers of muscle stress (cortisol and creatine phosphokinase [CK]), and antioxidant capacity (ferric reducing ability of plasma [FRAP]) were also assessed.

**Results:**

PB-H attenuated the decrease in peak torque observed in the placebo group from pre-exercise to 48 h (*p=*0.012) and 96 h (*p=*0.003) post-exercise. At 48 h post-exercise, PB-H reduced whole body and hamstring soreness (*p*=0.029) versus placebo. Chronic consumption of PB improved serum FRAP (*p*=0.039). As expected, serum cortisol and CK increased from pre- to post-exercise in all groups; however, by 96 h, cortisol and CK levels returned to pre-exercise levels following PB supplementation. At 96 h, the change in cortisol from pre- to post-exercise was significantly greater in placebo versus PB-H (*p*=0.039).

**Conclusion:**

These findings show that chronic consumption of PB improved antioxidant status, reduced markers of muscle stress, and promoted strength recovery post-exercise.

Recovery after exercise is important for athletes and recreationally active individuals. High-intensity exercise is associated with a decrease in power and overall performance during subsequent bouts of exercise (1–4). This negative impact on performance, measured as decreased muscle force production or strength loss, can be associated with delayed onset muscle soreness (DOMS) and is usually observed about 24 h post-exercise and can last as long as 5–7 days (1, 2, 5). The consensus of research suggests that decreased performance occurs in response to muscle microtrauma, which initiates a cascade of inflammatory and oxidative stress–related events. Accordingly, an intervention that could improve performance in the days following a high-intensity exercise bout would allow an athlete to train more frequently at higher intensities with less discomfort (6, 7).

Green tea and its catechin components are known to stimulate antioxidant activity by scavenging free radicals, inhibiting pro-oxidant enzymes, and stimulating antioxidant enzymes (8–11). In addition to potent antioxidant qualities, catechins and catechin-rich green tea extracts have both been shown to increase lipid utilization during exercise, which may allow for improved exercise capacity (12–15). Research suggests that theaflavins, a polyphenolic compound found in black tea, decrease both oxidative stress and inflammation that result from various physiological stressors. Theaflavins have been shown to reduce oxidative stress via free radical-scavenging (16–20). Most of the antioxidant and anti-inflammatory effects of theaflavins have been examined in relation to disease (17, 18, 21). There is limited research evaluating the effects of theaflavins, or their black tea source, on inflammation associated with exercise, oxidative stress, and the related systemic responses to exercise in humans. Green and black tea extracts have been researched independently; however, they have not been examined in combination to evaluate their complementary benefits for sports nutrition.

There is a noticeable absence in the marketplace for natural approaches with proven evidence to address the issues associated with muscle soreness post-exercise and the resulting performance decrements (22–24). Therapies such as non-steroidal anti-inflammatory drugs (NSAIDs), including aspirin, ibuprophen, and acetominophen, can be taken to temporarily reduce pain and inflammation associated with DOMS. However, these drugs do not address oxidative stress and performance strength loss linked to DOMS (25). Furthermore, in some individuals, prolonged or repeated NSAID usage can lead to nausea, stomach upset, and other digestive issues. Athletes involved in training sessions on successive days have traditionally relied on carbohydrate or protein/amino acid supplementation to replenish glycogen stores and stimulate muscle protein synthesis; however, data is still inconclusive regarding the benefits of carbohydrate/protein supplementation on subsequent exercise session performance and DOMS (26–28). A few recent studies have shown the ability of certain food extracts high in antioxidants to promote recovery following a bout of intense exercise (22, 23).

Polyphenolic constituents, including catechins and theaflavins, can provide both antioxidant and anti-inflammatory properties, which may be beneficial to athletes and non-athletes during competitive training and in general exercise regimens. Therefore, a clinical study was conducted to evaluate the efficacy of a novel ingredient, a proprietary polyphenolic blend (PB) containing catechins and theaflavins, on muscle performance, oxidative stress, and inflammation post-exercise.

## Methods

### Design

The current study used a randomized, double-blind, placebo-controlled design to investigate the influence of 13 weeks of supplementation with PB on exercise performance and recovery following an eccentric exercise challenge in recreationally active men. The longer supplementation period was chosen based on success in animal models (15). Recovery was assessed through measurement of strength losses (Biodex), perceived muscle soreness, and markers of muscle stress (lactate dehydrogenase [LDH], creatine phosphokinase [CK], cortisol and adrenocorticotropic hormones), inflammation (IL-6, IL-10, TNF-α), and oxidative stress (8-isoprostane, and ferric reducing ability of plasma [FRAP]) at pre- and post-exercise time points during the 13th week of supplementation. This investigation tested the hypothesis that 13 weeks of supplementation with PB would improve post-exercise recovery.

### Subjects

Men between the ages of 18–35 years, who were recreationally active in both cardiovascular and resistance training, yet not exercising for more than 6 h/week, were included. The training inclusion criteria required that participants be actively performing aerobic exercise and partaking in resistance training at least twice per week, for a minimum of 3 months.

Participants were excluded who were actively engaged in eccentric muscle training, downhill running, running >15 miles/week, or presented certain diseases or conditions such as HIV, hepatitis B and C, uncontrolled cardiovascular arrhythmias, chronic obstructive pulmonary disease, emphysema, diabetes, or unresolved orthopedic concerns. Additional exclusion criteria included a body mass index <18 or >30 kg/m^2^, use of tobacco products within the previous 12 months, and regular consumption of medications or over-the-counter therapies that might affect inflammation such as: green or black tea (≥8 oz./day), green or black tea supplements, cherry juice (≥8 oz./day), vitamin E (≥400 IU/day), vitamin C (≥1,000 mg/day), aspirin, corticosteroids, anabolic steroids, or NSAIDs. Finally, participants agreed to limit their consumption of alcoholic beverages to <3 drinks per day.

The study was conducted at the Pennington Biomedical Research Center (Baton Rouge, LA, USA). The Pennington Biomedical Center Institutional Review Board approved the protocol-specific procedures and informed consent. The study was conducted according to the International Conference on Harmonization Guidelines for Good Clinical Practice and the World Medical Association's Declaration of Helsinki. Written informed consent was obtained from all participants before any study procedures were performed. Demographics for evaluable participants are shown in Table 1. There were no differences between groups in any of the baseline characteristics or outcome measurements.

**Table 1:** Demographic characteristics for evaluable participants by treatment groups

	Treatment						
		
	High dose (*n*=12)		Low dose (*n*=12)		Placebo dose (*n*=13)		
				
	Mean	SEM	Mean	SEM	Mean	SEM	*p* *
Age (y)	24.33	1.54	21.67	1.12	22.69	1.00	0.325
Weight (kg)	78.39	3.57	80.02	4.36	79.27	2.27	0.948
Height (cm)	175.36	2.17	177.48	1.11	179.95	2.08	0.229
BMI (kg/m^2^)	25.47	0.98	25.31	1.18	24.49	0.62	0.730
Compliance to protocol (%)	96.94	1.58	96.48	1.11	97.67	0.91	0.784

* Means across groups were compared using an analysis of variance model.

### Interventions

**Fig. 1:**
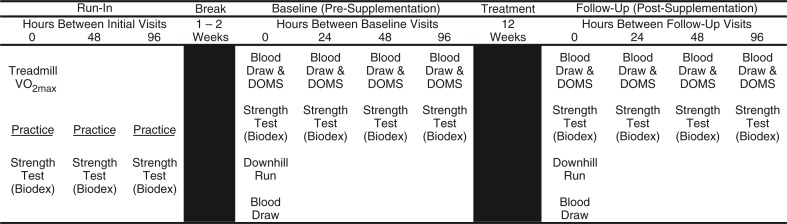
Overview of the clinical trial study design. The study design included three screening visits to facilitate equipment and protocol learning to remove learning effects and 4 days of criterion testing performed at baseline (pre-supplementation), following 12 weeks of supplementation. The post-supplementation testing period occurred during the 13th week of supplementation.

The current study design included participant recruitment, screening (run-in) to facilitate equipment and protocol learning and limit learning effects, and 4 days of testing performed at baseline (pre-supplementation) and following 12 weeks of supplementation (Fig. 1). The post-supplementation testing period occurred during the 13th week of supplementation, for a total of 13 weeks of supplementation.

#### Screening

Following a successful initial phone screen, candidates were invited to participate in 3 consecutive days of screening visits to practice the various study protocols and to complete a more in-depth health history and exercise questionnaire. During their first screening visit, all participants performed a treadmill running test to determine their maximal cardiorespiratory capacity, followed by a practice Biodex leg extension to evaluate muscle strength, which was repeated at two additional screening visits. Participants returned for baseline testing within 1–2 weeks of their final screening visit. In addition, participants completed an activity log throughout the screening period to document their habitual exercise regimen.

#### Baseline (pre-supplementation) and follow-up (post-supplementation) testing

Baseline and follow-up testing consisted of four visits per participant. Prior to all test visits, participants were asked to consume all medications as prescribed, drink at least 32 oz. of water within the previous 24 h, and abstain from the following: alcohol for 48 h, caffeine for 5 h, and vigorous exercise for 24 h prior to testing. Vigorous exercise was defined as those exercises outside of those reported as a part of their normal exercise routine as documented on their activity logs during the screening period. Participants consumed a regular meal 2–3 h before both the baseline and follow-up visits. Food records were completed 3 days prior to and during baseline testing, for a total of 7 days. These dietary records were then collected and redistributed during week 12 of the intervention. This meal was not standardized for all participants; however, each participant was instructed to replicate the type and quantity of the food during the follow-up testing in week 13 for consistency within the subject across the study. During baseline and follow-up testing, exercise was limited to only the study protocol and participants were asked to refrain from the use of ibuprofen, ice, or massage therapies. In addition, during the intervention period study participants were asked to complete daily activity logs to ensure that intensity or duration of their exercise regimen as captured during the screening period was unchanged.

At the first visit during the baseline and follow-up period upon participant arrival, blood was drawn for a complete metabolic panel and to analyze indices of muscle stress, inflammation, and oxidative stress. Current muscle soreness status was assessed by questionnaire before initiating any of the exercise protocol. Each participant was then given a 120 kcal snack (a commercially available cereal bar) and 8 oz. of water. At each time point during the follow-up testing period, participants also consumed a dose of their respective treatment on-site along with the snack. The rationale for the administration of the supplement during the follow-up testing period was to ensure the study design best reflected what an individual might expect following a chronic dosing regimen. Sixty minutes later, participants performed the Biodex leg muscle strength test and then performed the downhill treadmill run. Fifteen minutes after the downhill run, another blood sample was collected to examine the acute effects of the run on indices of muscle stress, inflammation, and oxidative stress.

During both the baseline period and the follow-up period, visits occurred at 24, 48, and 96 h after the downhill run. Upon arrival on the post-downhill run days, a blood draw was performed. Each participant was assessed for muscle soreness and provided with a light snack and water (plus treatment during the follow-up period). After 60 min, participants performed the Biodex leg muscle strength test.

#### Maximal cardiorespiratory assessment

A running protocol was utilized to determine VO_2_ max so that the downhill run could be conducted at a speed associated with 65% of the VO_2_ max. Testing was initiated on a treadmill at a controlled walking speed of 2.5 mph before progressing to 3.5 mph and then to 4.5 mph. Participants then used a series of hand signals to progress the speed. Each stage performed on a flat surface was 3 min in duration and proceeded as such until the participant achieved a respiratory exchange ratio of 1.0. Once the respiratory exchange ratio reached 1.0, the speed was held constant and grade was increased by 1% every minute until volitional fatigue. Treadmill tests were monitored with a 12-lead ECG (Quinton Q-Stress 12 Lead) for heart rate and oxygen consumption via open circuit spirometry using a Parvomedics TrueOne^®^ 2400 Metabolic System (Salt Lake City, UT, USA).

#### Downhill treadmill running protocol

The downhill running protocol consisted of running on a treadmill (Lode Valiant Treadmill ergometer) at a 10% decline for 40 min at a speed associated with ~65% of the VO_2_ max determined during the screening visit. Heart rate was monitored continuously (Polar). Downhill running protocols have been shown in previous studies to decrease post-exercise muscle function and increase markers of muscle stress (29–33).

#### Biodex leg muscle strength

All muscle strength tests were performed on a Biodex System 3 dynamometer (Biodex Medical Systems, Shirley, NY, USA). The Biodex was chosen because muscle groups of the upper leg would most likely be affected by downhill treadmill running. Prior to Biodex testing, participants completed a warm-up routine consisting of walking for 5 min on a treadmill at a slow pace. Participants performed three sets of quadriceps leg extensions on their dominant leg (identified during the screening visit and kept constant throughout the study) for 12 repetitions at 120 degrees/sec for evaluation of isokinetic extension. Subjects were instructed to kick (extend) as hard as they could and remain passive while the Biodex returned to the start position for each extension repetition. Each set was interspersed with 2 min of rest. Peak torque (highest repetition, N m), low torque (the average of the three consecutive lowest repetitions at the end of the set, N m), total work (N m), average power (Watts), and the fatigue index (%) were calculated for each set of exercises. Fatigue index was defined as the high torque (the average of the three consecutive highest repetitions at the beginning of the set) minus the low torque, divided by the high torque. For each of these variables, the average of three sets was used to represent a single visit.

#### DOMS

Muscle soreness was assessed using a 7-point Likert scale questionnaire for a variety of muscle groups including the gastrocnemius, hamstrings, quadriceps, gluteus maximus, lower back, abdominals, and the whole body (34). Participants were asked to rate their perceived level of muscle soreness at rest as 1) no pain, 2) dull ache, 3) slight pain, 4) moderate pain, 5) painful, 6) very painful, or 7) severe pain.

#### Blood chemistry

A comprehensive metabolic panel was conducted to evaluate the safety of and tolerance to the dietary supplement. Blood was also evaluated to examine potential mechanisms of action of the treatment through the evaluation of various markers of inflammation (IL-6, IL-10, TNF-α), oxidative stress (8-isoprostane, FRAP), muscle stress (LDH, CK), and muscle catabolism (cortisol and adrenocorticotropic hormone). Whole blood was collected and serum was isolated, banked, and stored at −80°C to be analyzed in a batch at the end of the study.

Accordingly, IL-6, IL-10, and TNF-α were assayed from frozen serum samples using an immunoassay with fluorescent detection (Luminex Labmap 100, Lincoln, St. Charles, MO, USA). LDH and CK were analyzed from frozen serum using an immunoassay with chemiluminescent detection (Beckman Coulter DXC600, Brea, CA, USA). Cortisol and adrenocorticotropic hormone were analyzed using immunoassay chemiluminescent detection on an Immulite 2000 (Siemens, Tarrytown, NY, USA). FRAP, which measures the antioxidant capacity of the blood, was measured by a colorimetric assay (Beckman Coulter DXC600); and 8-isoprostate was measured by enzyme immunoassay (Tarrytown, NY, USA).

#### Treatment

The two active study groups consumed different doses of PB (Kemin Foods, L.C., Des Moines, IA, USA), a blend of water-extracted black and green tea (*Camellia sinensis*) containing a minimum of 40% total polyphenols, 1.3% theaflavins, 5–8% epigallocatechin-3-gallate (EGCG), 7–13% caffeine, and 600 ppm manganese and formulated under good manufacturing practices. The nutritional analysis for the polyphenol blend extract is shown in Table 2. The study product was encapsulated in gelatin capsules and packaged in light-resistant plastic bottles (Five Star Compounding Pharmacy, Des Moines, IA, USA). The product lots were tested for toxins including heavy metals, pesticides, and excipients. Stability of the capsules was confirmed throughout the study period by measurement of the active components.

**Table 2:** Nutritional analysis of polyphenolic blend

Nutrient	Amount per 1 g
Calories	3.65 cal
Calories from fat	0.02 cal
Total fat	0 g
Saturated fat	0 g
Trans fat	0 g
Cholesterol	<0.01 mg
Sodium	3.72 mg
Total carbohydrates	0.62 g
Dietary fiber	0.1 g
Sugars	0.01 g
Protein	0.28 g
Calcium	0.69 mg
Iron	0.07 mg

After baseline testing, all participants were randomly assigned to one of three treatments matched for color and capsule size: 1) a placebo treatment with 4 capsules/day containing 500 mg microcrystalline cellulose excipient, 2) a low-dose PB extract (PB-L; 1,000 mg/day), 4 capsules/day each containing 250 mg of PB extract and 250 mg of microcrystalline cellulose, or 3) high-dose PB (PB-H; 2,000 mg/day), 4 capsules/day each containing 500 mg of PB extract.

To consume the required dose, participants ingested 2 capsules twice per day with a meal. Participants returned at the end of each month to obtain new supplements, and capsules were counted against a known quantity of administered treatment capsules in order to assess compliance. *A priori*, the evaluable group was defined as those individuals who were >80% compliant with study product consumption. Participants were required to maintain the same exercise and dietary habits during the intervention as reported during screening and were contacted on a weekly basis by phone or email to ask about any adverse events, and encourage compliance and maintenance of exercise and dietary habits.

### Statistical analysis

Analysis of the data for all variables during baseline testing (pre-supplementation) revealed no differences between groups. Therefore, each outcome variable value used to assess patient recovery was transformed to reflect the change from pre-exercise to post-exercise during the post-supplementation period (following 12 weeks of supplementation). Each transformed variable was analyzed using a mixed model repeated measures (MMRM) analysis of variance containing terms for treatment, visit, and the treatment×visit interaction. Least squares means were generated from the model for both main effects and the interaction. Finally, significance levels were determined for main effects, interactions, and pairwise comparisons of each treatment group against the placebo.

One additional analysis was performed to compare serum antioxidant status as measured by FRAP. Using a MMRM model to assess change values between corresponding time points preceding and following the 12-week supplementation period, a contrast from the MMRM model was formed to compare the pooled treatment groups to the placebo between the first available FRAP measurement prior to 12-week supplementation and the first available FRAP measurement post–12-week supplementation. The statistical analysis was completed by Summit Analytical, LLC (Denver, CO, USA). All data are presented as mean±standard error of the mean (SEM) and all tests of significance were completed at α=0.05, two-tailed.

## Results

### Subjects

**Fig. 2:**
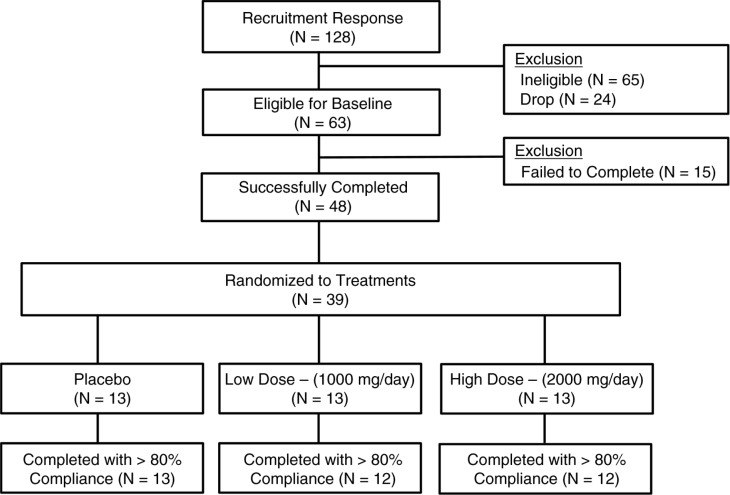
CONSORT diagram. Participant screening through study completion is shown for all study participants. Compliance was prospectively set at>80% over the 13-week supplementation period.

One hundred twenty-eight participants were screened, of which 63 were eligible for baseline measurements based on inclusion and exclusion criteria, 48 completed baseline testing, and 39 were randomized. Thirteen individuals from each group completed the intervention period; however, the study physician withdrew one individual from the PB-H group during the follow-up testing period due to clinically elevated CK levels post-exercise. In addition, one individual from the PB-L group was removed from the analysis due to the *a priori* decision not to include individuals with compliance less than 80%. The final evaluable number for analyses was 12 per group for the PB-L and PB-H groups and 13 in the placebo group. A CONSORT schematic outlining the overall study is provided (Fig. 2).

### Post-supplementation Biodex leg muscle strength

**Fig. 3:**
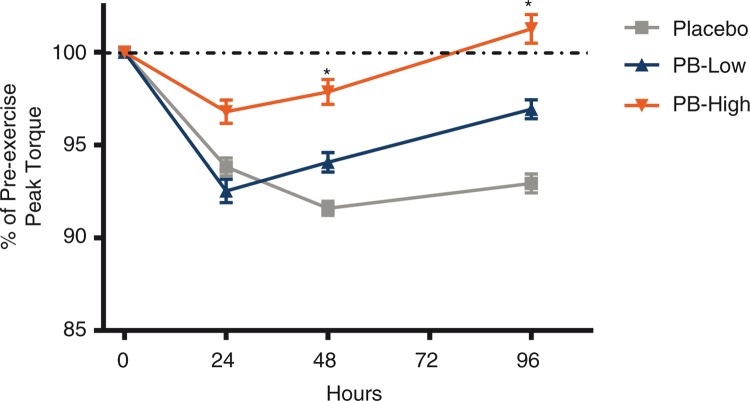
Biodex peak torque following chronic supplementation. Biodex peak torque is shown at 24, 48, and 96 h post-exercise following supplementation with a polyphenolic blend (PB) (PB low, 1,000 mg/day; PB high, 2,000 mg/day) or placebo. Dashed line indicates the strength reported pre-exercise. Data are presented as mean±SEM. *p<0.05 represents the between group difference for the change from pre-exercise in the PB high versus the change from pre-exercise observed in the placebo group.

The overall analysis of variance model for peak torque was significant for treatment. Following 12 weeks of supplementation, peak torque was significantly decreased within the placebo group at 24, 48, and 96 h compared to pre-exercise peak torque (*p*=0.0013, *p*<0.0001, *p*=0.0003, respectively). Participants supplemented with PB-L for 12 weeks also showed significantly decreased peak torque at 24 and 48 h post-exercise (*p*=0.0004 and *p*=0.010, respectively) compared to their pre-exercise peak torque; however, at 96 h, the peak torque levels were not significantly different than their pre-exercise values. Similarly, participants supplemented with PB-H showed no significant decreases in the peak torque values at any time point (24, 48, or 96 h) post-exercise compared to pre-exercise. Additionally, the decrease in peak torque in the placebo group was significantly greater than the decrease observed in the PB-H group at both 48 and 96 h post-exercise (*p*=0.012 and *p*=0.0031, respectively, Fig. 3). No differences were identified in low torque, total work, average power, or fatigue index following supplementation with PB versus the placebo group.

### Post-supplementation DOMS

Significant improvements in DOMS were observed for the PB-H treatment group at 48 h post-exercise following the 12 weeks of supplementation compared with the placebo, whereas only trends were observed in PB-L treatment group. Subjective muscle soreness decreased in both the hamstrings (*p*=0.029) and whole body (*p*=0.029) at 48 h post-exercise for participants administered PB-H. No differences in muscle soreness were identified in gastrocnemius, gluteus maximus, lower back, or abdominals following supplementation with PB versus placebo.

### Blood measurements

**Fig. 4:**
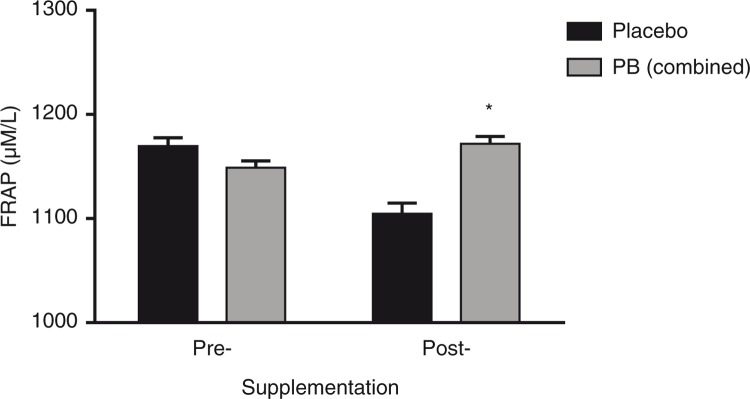
Ferric reducing antioxidant power (FRAP) following chronic supplementation. Pre-exercise FRAP values are shown pre- and post-supplementation with either a polyphenolic blend (PB) or a placebo. Mean for treatment represents the combined treatment groups (PB low, 1,000 mg/day; and PB high, 2,000 mg/day). Data are presented as mean±SEM. *p<0.05 between group difference for the change (post-supplementation from pre-supplementation) in the combined PB groups versus the same change in the placebo group.

The chronic change in serum antioxidant status following 13 weeks of PB supplementation compared to pre-supplementation values, as measured by FRAP, was significantly different than the change observed in those participants receiving the placebo (Fig. 4, *p*=0.039).

**Fig. 5:**
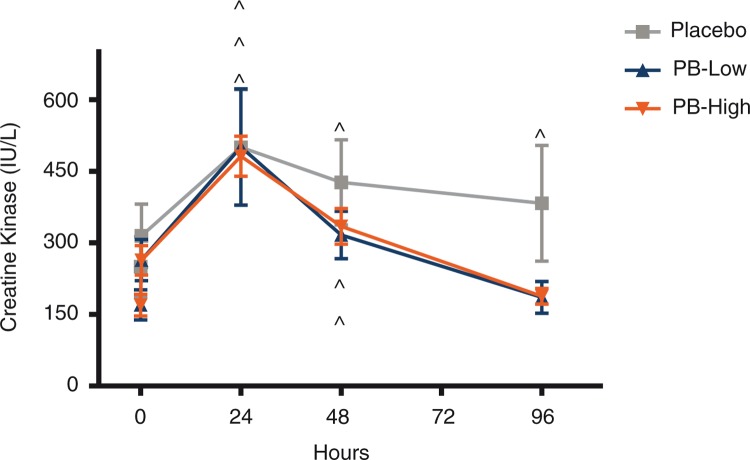
Creatine kinase (CK) levels following chronic supplementation. CK levels are shown following supplementation with a polyphenolic blend (PB) (PB low, 1,000 mg/day; PB high, 2,000 mg/day) or placebo. CK was measured pre-exercise, and 15 min and 24, 48, and 96 h post-exercise. Data are presented as mean±SEM. ^∧^ *p*<0.05 versus pre-exercise level within each treatment group.

Post-supplementation, as expected, participants supplemented with the placebo had significantly increased CK values at 24 h (*p*<0.001), 48 h (*p*=0.001), and 96 h (*p*=0.010) post-exercise in comparison to pre-exercise levels, with CK never returning to corresponding pre-exercise levels within the entire testing period. However, although participants supplemented with both PB-H and PB-L showed significantly greater CK values post-exercise at both 24 h (PB-H *p*<0.001; PB-L *p*<0.001) and 48 h (PB-H *p*<0.002; PB-L *p*=0.006) as compared to pre-exercise values, by 96 h CK values in both PB supplemented groups were not significantly different from and had returned to pre-exercise CK levels (Fig. 5).

**Fig. 6:**
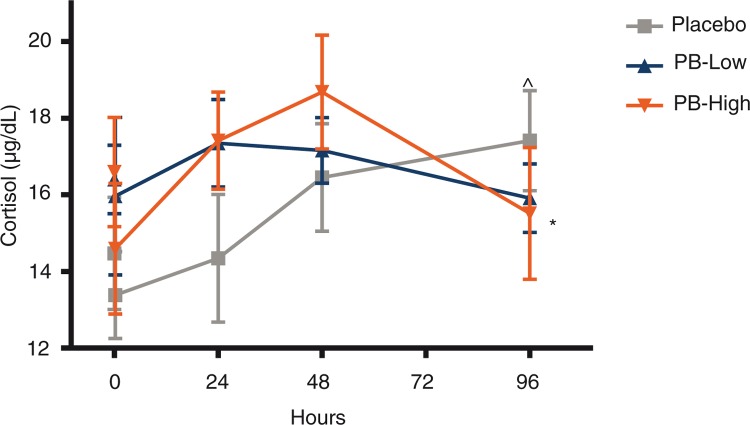
Cortisol levels following chronic supplementation. Cortisol levels are shown following chronic supplementation with a polyphenolic blend (PB) (PB low, 1,000 mg/day; PB high, 2,000 mg/day) or placebo. Serum cortisol was assessed pre-exercise, and 15 min and 24, 48, and 96 h post-exercise. Data are presented as mean±SEM. ^∧^ *p*<0.05 versus pre-exercise value within treatment group. *p<0.05 between group difference for the change from pre-exercise in the PB high versus the change from pre-exercise observed in the placebo group.

Post-supplementation cortisol levels continued to increase in the placebo group in comparison to the corresponding pre-exercise levels and remained statistically higher at 96 h post-exercise (*p*=0.026). These observed increases in cortisol within the placebo group were significantly greater than those observed in the PB-H group (*p*=0.039, Fig. 6). No changes were identified in serum cytokines, 8-isoprostane, LDH, or adrenocorticotropic hormone following chronic supplementation with PB versus the placebo group.

## Discussion

This randomized, double-blind, placebo-controlled clinical study demonstrates that 13 weeks of supplementation with PB improves post-exercise recovery. Supplementation with PB for 13 weeks resulted in significant improvements in serum antioxidant status accompanied by a quicker recovery from a muscle damaging exercise protocol as compared to the placebo treatment. PB supplementation resulted in a significant attenuation of peak torque losses over 96 h post-exercise, a quicker return of cortisol and CK to pre-exercise values over the 96 h evaluation period, and significantly lower muscle soreness ratings 48 h following the downhill run. These results indicate that supplementation with PB can help minimize exercise-induced performance decrements, thereby allowing individuals to train harder and/or more frequently. Prior to the current study, no human clinical data existed supporting the ability of tea polyphenols to attenuate post-exercise losses in muscle strength. These findings are supported by a recently published study by Haramizu et al. which found that catechin supplementation of male ICR mice for 3 weeks attenuated the downhill running-induced decrease in wheel-running activity by 35%, increased running endurance by 13%, and enhanced recovery from loss of muscle contractile force concurrent to reduction in markers of muscle damage and oxidation (35). These animal data suggest that supplementation with tea polyphenols may diminish post-exercise muscle damage, hasten recovery, and improve physical performance. The animal study conclusions support the findings of the current study which provides the first solid evidence that supplementation with a proprietary blend of tea polyphenols, including catechins and theaflavins, may help athletes overcome post-exercise strength losses brought on by the intense exercise perhaps through the suppressive action of polyphenols on oxidative stress and inflammation. These findings have critical importance to establishing a scientifically proven and clinically relevant nutritional ingredient that facilitates post-exercise recovery and may have positive implications on athletic performance.

The literature shows that following eccentric exercise reactive oxygen species (ROS) can activate cell signaling mechanisms resulting in a downstream cascade of events, including elevated serum cortisol and CK (32, 36), as observed in the current study. CK levels post-exercise vary from less than 1,000 IU/L for a mild muscle damaging protocol to greater than 10,000 IU/L for severe muscle damaging protocols (25). Data is more limited for cortisol levels post-exercise, but concentrations 1.1 to 2 times the pre-exercise levels are reported in the literature (37). The reported presence of ROS in muscle tissue post-exercise suggests that antioxidant supplementation may minimize the downstream effects and reduce DOMS after exercise (36). However, despite numerous studies conducted on the use of antioxidant vitamins C and E, either administered in combination or separately, there is no consensus in the literature that these antioxidants are effective in reducing the loss of strength, power, or the onset of DOMS associated with exercise (37–40). Polyphenols are a promising group of antioxidants that have been evaluated by researchers (15, 16, 22, 41–43). A number of studies indicate the effectiveness of polyphenols, such as catechins, as antioxidants through suppression of NF-κB activation, a key initiator of the cascade of cell signaling, thus showing the potential of catechins to attenuate the activation of exercise-induced inflammation and soreness (44, 45).

In the present study, the downhill run was intended to trigger oxidative stress and the associated post-exercise cascade of events. This was achieved in all groups as evidenced by losses in strength, increases in both CK and cortisol, and higher muscle soreness ratings immediately post-exercise. CK significantly increased post-exercise peaking at 24 h post-exercise, with no differences between groups, suggesting similar levels of muscle damage and oxidative stress. However, at 96 h post-exercise, the placebo group had significantly higher CK values, whereas both the PB supplemented groups returned to pre-exercise levels, suggesting a quicker CK clearance and enhanced muscle recovery.

Similar findings were also obtained for cortisol, corroborating the effects seen with CK following chronic PB supplementation. Cortisol responses are directly related to exercise intensity and duration, increasing post-exercise (46). Although no between group differences were identified at earlier time points, circulating cortisol in the placebo group remained significantly higher at 96 h compared to the PB-H group. Studies have shown that supplementation with either N-acetyl cysteine or EGCG alone in active men did not affect cortisol levels over a 72 h post-exercise recovery period (47). However, supplementation with theaflavin-enriched black tea extract has been shown to lower overall cortisol secretion within the first h post-exercise (22). In the current study, the subsequent continued exercise on days 1, 2, and 4 may have allowed differences in cortisol and oxidative stress responses to be identified. Due to of the catabolic nature of cortisol, the ability of PB to significantly reduce cortisol secretion post-exercise may facilitate improved recovery and decreased muscle stress. Although initial elevations in cortisol may be beneficial, prolonged elevations in cortisol can lead to chronic inflammation. Excessive cortisol secretion in response to exercise may contribute to overtraining (3).

One of the major deterrents to performing subsequent activities after an intense bout of exercise is DOMS. Although an eccentric exercise was used as a trigger to inflict muscle soreness in the current study, DOMS is often felt after vigorous exercise. Compared to placebo, PB-H supplementation significantly blunted the increase in both whole body and hamstring muscle soreness ratings at 48 h post-exercise. The individuals in the current study were not sedentary, reporting (amongst all groups) approximately 5 h of activity per week at screening. The more active subjects used in the current study may have displayed enhanced tolerance to DOMS and blunted physiological responses after training, thereby impairing our ability to detect greater changes in DOMS and markers of muscle damage or catabolism (48).

After an intense or unaccustomed exercise bout, secondary damage associated with inflammatory processes may occur over several days following the initial insult (49). Research has focused on nutritional strategies aimed at reducing the oxidative stress and inflammation traditionally associated with fatigue and impaired recovery post-exercise. Although changes in CK, cortisol, and muscle soreness were observed after the downhill run, several markers of inflammation, IL-6, IL-10, TNF-α, and isoprostanes did not significantly increase after the downhill run. However, this difficulty or inability to detect changes at the circulating level suggests future work should incorporate muscle biopsies. An additional confounding factor is that trained subjects often display attenuated stress response to exercise challenges, which has been attributed to several physiological adaptations including increased heat shock protein levels or an enhanced antioxidant capacity (49). In addition, the ability to accurately assess changes may have been missed due to the time points chosen for analyses, which were not ideal for all biomarkers (15 min, and 24, 48, and 96 h post-exercise).

The current study also demonstrated that chronic PB supplementation increased FRAP over placebo, which is in agreement with other studies showing increased FRAP in exercising populations after supplementation with green tea (600 mL/day) or grape extract (50–52). Grape extract was able to limit the reduction of FRAP in elite athletes during training compared to the placebo group (52). Green tea extract significantly increased plasma FRAP in the context of resistance training workouts after 14 days and showed a trend after 7 days (50, 51). Although the active constituents of PB were not directly measured in blood in the current study, the significant chronic increases in serum FRAP in the treatment groups over placebo support that the supplement was bioavailable. The ability to increase serum antioxidant status may minimize the oxidative stress-induced cell signaling post-exercise, thus positively affecting biomarkers (such as CK and cortisol) and ultimately muscle soreness and performance during the recovery period.

Strengths of the current study include a randomized, double-blind, placebo-controlled study design and supplementation intake compliance at greater than 95%. The performance strength testing used is regarded as the best functional marker of muscle damage aside from muscle biopsy collection which is both invasive and expensive (25). Furthermore, participants were introduced to the protocol through three familiarization sessions during the screening period to minimize any potential learning effects. Still another asset of the current study is that strength recovery, blood biomarkers, and muscle soreness were all measured simultaneously. Arent et al. (22) also showed that supplementation with 1,760 mg black tea extract per day for 9 days containing 700 mg theaflavins improved peak and average power during bouts of high-intensity cycling, but unfortunately the authors did not evaluate strength recovery concurrent to muscle soreness and cortisol measurements. The simultaneous measurements carried out in the current study revealed that the greatest strength recovery corresponded to the time point when cortisol and CK returned to pre-exercise levels suggesting a possible mechanism of action for the enhanced recovery and performance.

Limitations of the study include that the downhill treadmill run protocol may not have been as intense of a muscle insult as anticipated, at least for these participants, and hence, likely did not result in high levels of muscle damage. The downhill run exercise stimulus in the current study can be regarded as mild to moderate muscle damaging due to the less than 20% reduction in muscle strength post-exercise and peak CK values that were less than 1,000 IU/L (25). Strategies to increase the muscle stress in future work could include a steeper treadmill slope, concurrent monitoring of the O_2_ and CO_2_ gases to ensure all participants were consistently at 65% VO_2_ max, or the use of sedentary participants. Nevertheless, the ability to see significant differences with PB supplementation even in the absence of an intense muscle damaging insult may benefit a wide range of active individuals.

This randomized, double-blind, placebo-controlled clinical trial demonstrates that 13 weeks of supplementation with PB, a proprietary blend of polyphenols including catechins and theaflavins, improves antioxidant status alleviating oxidative stress associated with exercise. In the current study, supplementation with PB resulted in reduced muscle soreness and improved post-exercise recovery as assessed through functional and objective measures of muscle stress.
